# Review on Acoustic Droplet Vaporization in Ultrasound Diagnostics and Therapeutics

**DOI:** 10.1155/2019/9480193

**Published:** 2019-07-14

**Authors:** Ksenia Loskutova, Dmitry Grishenkov, Morteza Ghorbani

**Affiliations:** ^1^Department of Biomedical Engineering and Health Systems, KTH Royal Institute of Technology, SE-141 57 Huddinge, Sweden; ^2^Mechatronics Engineering Program, Faculty of Engineering and Natural Science, Sabanci University, Istanbul 34956, Turkey

## Abstract

Acoustic droplet vaporization (ADV) is the physical process in which liquid undergoes phase transition to gas after exposure to a pressure amplitude above a certain threshold. In recent years, new techniques in ultrasound diagnostics and therapeutics have been developed which utilize microformulations with various physical and chemical properties. The purpose of this review is to give the reader a general idea on how ADV can be implemented for the existing biomedical applications of droplet vaporization. In this regard, the recent developments in ultrasound therapy which shed light on the ADV are considered. Modern designs of capsules and nanodroplets (NDs) are shown, and the material choices and their implications for function are discussed. The influence of the physical properties of the induced acoustic field, the surrounding medium, and thermophysical effects on the vaporization are presented. Lastly, current challenges and potential future applications towards the implementation of the therapeutic droplets are discussed.

## 1. Introduction

The dynamic behavior of bubbles and droplets has been investigated since 19th century, but their potential in medical applications was first reported by Gramiak et al. [1]. During the echocardiagraphic recording of the aorta root, they noted a strong echo from its lumen that had not been presented previously. It was later discovered that the origin of the increased echo signal is cavitation bubbles that enter the bloodstream during the injection of agitated saline [2]. This discovery led to the development of various formulations of gas-filled microbubbles (MBs) that could act as ultrasound contrast agents, allowing gain of novel functional and morphological information.

Acoustic droplet vaporization (ADV) was discovered in the 1990's and is the physical process of phase change of a superheated liquid to gas due to pressure variations in an ultrasonic wave. Following ADV, microcapsules with a liquid core can be converted to gas core MBs and act as the ultrasonic contrast agents. The oscillations of the ADV-generated bubble result in a scattered acoustic pressure field, which can be detected by a receiving transducer. At low pressures the oscillations are linear; however, the MB responses are nonlinear at pressure amplitudes used in the most medical applications. Further increases in acoustic pressure lead to bubble destruction and release of gas content and/or therapeutic payload [3].

By benefiting from the broad potentials of ADV in medical applications, with consideration of its capability in localized noninvasive energy exposure, it is possible to utilize its effect in targeted drug delivery [4, 5], embolotherapy [6], ultrasound imaging [7–10], transdermal noninvasive drug delivery [11], theranostics [12, 13], thermal therapy [14, 15], and histotripsy [16, 17].

The objective of this review paper is to provide the reader with background knowledge of the fundamental concepts and recent tools necessary to implement ADV. Accordingly, various capsule designs are presented in Section 2. The physical and chemical properties of the therapeutic droplets as well as their manufacturing processes are considered in detail in this section. The ADV efficiency as a function of various parameters is presented in Section 3. The current and new frontiers progressing applications of ADV are extensively discussed in Section 4. Finally, the present and potential future applications of ADV are included in Section 5.

## 2. Capsule Design and Production

Today, there are many different capsule designs available that have been investigated for different applications. They differ in terms of shell and core materials, size, and possible additional particles and/or ligands that enable a wide range of applications in both diagnostics and therapy.

Phase-change contrast agents (PCCAs) have gained increased interest in recent years due to their versatility [4, 19, 20]. They have a perfluorocarbon (PFC) core, which is converted to gas phase when the peak negative pressure (PNP) amplitude of the induced acoustic field increases above a certain threshold. Depending on the PNP amplitude, PCCAs act as linear and nonlinear scatterers or disrupt completely.

**Figure 1:**
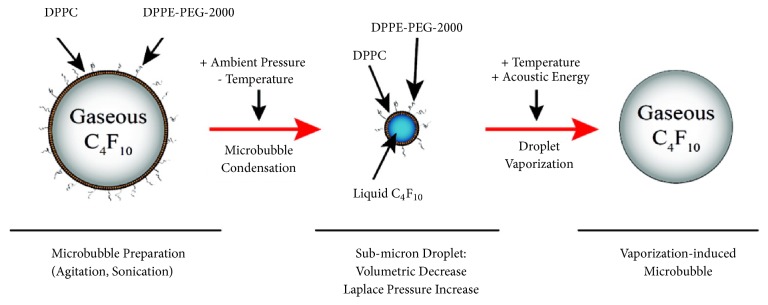
The manufacturing process of PCAs using the “condesation” method. The capsule with gas content is exposed to lowered temperature and increased ambient pressure, forcing the core material to phase transit to a liquid. The perfluorocarbon core is superheated, so the nanodroplet will be stable. The core will undergo phase transition back to gas after ultrasound exposure or if the temperature is significantly increased above the boiling point. Reprinted (adapted) with premission from [18]. Copyright 2011 American Chemical Society.

A common method to produce PCCAs is to mix the shell and core materials, and subsequently form the final droplet by mechanical agitation [5, 21, 22]. This approach is easy, fast and cheap, and can produce sub-micron droplets if the PFC has a boiling temperature comparable to normal room temperature. In recent years, PFCs such as decafluorobutane (DFB), with a boiling point below the physiological temperature of humans, have gained interest since the ADV pressure magnitude threshold for capsules with these core material is lower* in vivo* compared to capsules with other types of PFCs. A technique called “condensation method” has been used in several studies to produce PCCAs containing highly volatile PFCs such as DFB. A general overview can be seen in Figure 1 [23]. In short, this method works by first producing precursor gas bubbles as described previously and then condenses the inner core material by keeping the temperature low and increasing the pressure in the sample. It is possible to use these techniques to design droplets for drug delivery by cross-linking the shell materials to a molecule of interest, mixing a drug of choice with the PFC, or adding nanoparticles to the PFC liquid [24, 25], ultrasound-triggered molecular imaging [26], and dual-modality contrast imaging [8].

The limitation of the condensation method is that the droplets can be polydisperse, which causes difficulties when trying to optimize the acoustic field parameters. A possible alternative, which has drawn huge attention, is microfluidics. Martz et al. [27, 28] have developed a microfluidic setup to produce lipid-shelled PFC droplets, which can be acoustically triggered. The size of final droplets depended only on the flow rate of the lipid solution, making it easy to fine tune the diameter of the droplets to suit a certain application. One drawback of this technique is the very low production rate compared to other techniques [27]. However, it is still an interesting approach that could be used when monodispersity is highly demanded. An alternative microfluidic setup has been recently designed by Teston et al. [29], where the size of the final droplets depended on interfacial tension forces rather than flow rates. It was shown that the production rate is increased by a factor of 4, and further development of this technique has potential to become a viable manufacture method in the future. This and other microfluidic setups may be used to generate double emulsions [30], which can be an interesting new approach to drug-loaded capsule production in cases, where the active agent is highly hydrophilic. Double emulsions for transport can also be produced by sonication [31]; however, one major disadvantage of this approach is low stability during storage. Applications of novel shell materials can help improving the shelf life of drug-loaded double emulsions for easier handling in healthcare facilities.

Capece et al. [32] reported a novel strategy for generation of biocompatible polymer-shelled MBs from biodegradable vesicles that underwent ultrasound irradiation via ADV. They produced PFC core droplets covered with surfactant layer, which was attached to a hydrophilic polymer grafted with a vinyl moiety. At the final stage, the processed vesicles were adjoined to a radical photopolymer. The fabricated vesicles experienced phase transition as a result of ADV during the ultrasound irradiation. The authors reported a twofold increase and recovering in the mean diameter for transition of the droplets to MBs during the ultrasound exposure and dissipation, respectively. Moreover,* in vitro* experiments with harmonic modality showed a safe threshold for the medical ultrasound platform during the ADV evolution. The authors claimed that these polymer-shelled MBs may be utilized on designing the theranostic devices according to their imaging and therapeutic potentials.

Acoustically active droplets with a shell, cross-linked with a certain protein, can be used to targeted cell-surface receptors. Sheeran et al. [33] produced submicron sized droplets with a lipid shell and a DFB core using the condensation method. The droplets were designed to target *α*_*v*_*β*_3_ integrins by cross-linking a cyclic RGD peptide to one of the lipid compounds. Human umbilical vein endothelial cells have been previously shown to overexpress the *α*_*v*_*β*_3_ integrins and were therefore chosen for testing. A proprietary pulse sequencing technology was used before and after ultrasonic actuation to provide information about the change in signal intensity. The results showed that the droplets are successfully vaporized when exposed to the activation pulse. The intensity increase for targeted samples was statistically significant, while this was not the case for nontargeted. However, in a study performed by Rojas et al. [34], the signal intensity was stronger from targeted MBs than for targeted droplets. Additionally, both formulations remained in the tumor tissue after ultrasound exposure, which was expected for NDs but not for MBs. Further investigations are needed to clarify the reason behind this unforeseen result and to explore the potential of these droplets in diagnostics of various diseases both in the vascular system and in the intertissue.

Recently, a multimodal phase-change contrast agent was developed by Lin et al. [8] that enables contrast enhancement in three ways: (1) photoacoustic imaging, (2) ultrasound imaging with optical activation, and (3) ultrasound imaging with acoustic activation. DFB was used as core material to decrease the boiling point and to reduce undesirable bioeffects. Cyanine7.5 with absorption peak at 788 nm was used as optical absorber. The results showed that Cy-droplets could act as a new dual-triggerable and dual-modality submicron PCCA, with no significant difference in the results between the employed and blank droplets that did not contain an optical absorber. When studying the photoacoustic signals and imaging contrast of Cy-droplets and controls, the Cy-droplets were the only ones producing a good signal with high enough signal-to-noise ratio (≈10). The developed formulation could be used in applications requesting dual modality high selectivity, e.g., cancer molecular imaging and targeted drug delivery.

Although the most common design for ultrasound contrast agents is droplets with a PFC core, new design options have come up in recent years. One of them is mesoporous silica nanoparticles (MSNs) developed by Jin et al. [7]. The MSNs used in this study were produced from precursor mesoporous silica nanoparticles made according to a scheme from a different study [35] and then covered with two types of silanes: superhydrophobic trimethylchlorosilane (for F-MSN) and fluorodecyltriethoxysilane (for M-MSN). The size, hydrophobicity level, and the effect of bubble nuclei located in the pores of the MSNs were assessed. Nonporous Stöber silica nanoparticles coated with silane and mesoporous nanoparticles without the hydrophobic coating were used as control. The results showed that the hydrophobic MSNs had significantly higher contrast enhancement when exposed to acoustic fields with mechanical index (MI) ranging from 0.2 to 1.2, compared to the control particles. Although a higher concentration of hydrophobic MSNs was required in serum than in water due to increased viscosity and lower surface tension, they were a viable option.

Yildrim et al. [36] developed a similar type of MSNs, but with phospholipids to stabilize the surface. They showed that there was significantly less heating in the tumor spheroid exposed to MSNs and high intensity focused ultrasound (HIFU) even at very high MI = 1.6 (at 1.1 MHz), when significant cell damage was observed. These results suggest that MSNs could be effective for tumor ablation when it is required to maximize mechanical damage and at the same time diminish thermal side effects such as skin burns and enteroparalysis.

## 3. Activation of Acoustic Droplet Vaporization

### 3.1. Bubble Design Effects on Bubble Dynamics during Activation

ADV is a complex phenomenon, where many different parameters can affect the phase-change progression. It is essential to understand how the process evolves in order to be able to develop safe and efficient ultrasound pulse sequences for droplet vaporization. One question that has been of interest is to determine the location of the initial nucleation site. A study performed by Li et al. [37] showed that the gas nucleation site is always located inside the droplet at the acoustic parameters 7.5 MHz and 3-15-cycle single pulses at various PNP [45]. Occasionally, a second nucleation site was observed on the direction of ultrasound propagation shortly after the formation of the initial site, which was consistent with previous studies in this field [46, 47]. Further studies are necessary to demonstrate if this applies to low-boiling-point PFCs and lower acoustic frequencies.

The structure of the droplet and its composition have been shown to affect the vaporization event. Droplets with a low-boiling-point PFC core show complex behavior. It has been shown that, after exposure to a short vaporization, they go through overexpansion with subsequent damped oscillations until the final diameter is reached. Since there are few theoretical models that are able to predict this behavior to a satisfactory degree, this presents an obstacle when it is required to optimize transducer parameters. Doinikov et al. [39] tried to overcome this fact by proposing a new model taking the parameters into consideration, which were previously oversimplified, and then compared the model with experimental results for octafluoropropane (OFP) and DFB droplets of various sizes. They derived the model from a combination of Navier-Stokes equation and the equation of continuity under the following assumptions:

- The evaporation rate change throughout the vaporization process;
- The bubble overexpand and then oscillate until a final diameter is reached, which is smaller than the maximum observed;
- The vapour bubble is located and growing from the center of the PFC droplet.

The error between theoretical and experimental results was sufficiently low for both OFP and DFB droplets when exposed to 8 MHz 2-cycle pulses at 500 kPa. Maximum error was observed for perfluoropentane (PFP) droplets exposed to a six-cycle, 3.5 MHz, and 4.5 MPa pulse, and it was presumed that this inaccuracy was because the presence of gas fraction inside the initial vapor bubble for the driving pulses was not taken into account by the model.

Due to the overexpansion and damped oscillations, droplets with highly volatile cores produce a unique acoustic signal that can be differentiated from MB contrast agents [41]. In short, 200 pulse signals measured at each PNP magnitude, wall-filtered in a way similar to conventional Doppler ultrasound, converted to the frequency spectrum, compensated for the frequency-dependent sensitivity of the receiving transducer, bandpass filtered with a 100-order Butterworth finite impulse response filter, and finally integrated in the same frequency band to obtain the final signal. This output could have potential applications in dual-modality imaging.

Aside from the core material, the shell has substantial affect on the bubble dynamics as well. Reznik et al. [48] observed that the acoustic behavior of a coated ADV-generated bubble is different from an uncoated bubble due to the presence of a viscoelastic shell material. Furthermore, the type of viscoelastic material affects the droplet efficiency. Lipid-coated bubbles were shown to compress but hardly expand because of the buckling of the shell [49], whereas fluorosurfactant-coated bubbles below the resonant size preferred expansion-dominated oscillations [48].

### 3.2. Thermophysical Effects on the Efficiency of the Acoustic Droplet Vaporization

Apart from the droplet characterization and ADV effectiveness on the applied region, the physics of ADV and the acoustic characterization are essential parameters in the initiation of the vaporization. This part, which is related to acoustic wave physics, implies that ADV is mostly dependent on ultrasound pressure, frequency, and temperature. Miles et al. [38] tried to find incident negative pressure—called an ADV threshold—which is necessary for the induction of nucleation. It was successfully shown that the negative pressure required for the nucleation prior to collapse can be determined via perturbation analysis of a compressible inviscid flow around a droplet for various frequencies and diameters. The model is only valid though for cases when the pressure differences between the inner and outer regions of the droplet are not so large. In addition, the ADV threshold strongly depends upon the transducer shape and focal working condition, lateral nonlinear diffusion, and the droplet composition itself.

### 3.3. The Surrounding Medium Effect on the Acoustic Droplet Vaporization Efficiency

The fluid medium which constitutes the droplet emulsion and the surrounding fluid constructs a significant field within ADV. In this regard, there are many studies which illustrated that the diameter of the droplets subjected to the acoustic waves undergoes a significant expansion by factor 5 to 6 [50–52]. The expansion ratio increases once the droplets experience an emulsification with an aqueous liquid as a result of the dissolved gas diffusion in or out of the droplets. Radhakrishnan et al. [53] investigated the variations of the dissolved oxygen content of phosphate-buffered saline (PBS), which diluted with PFP droplets before and after ADV implementation. Although the previous investigations supported the idea of the reduction of the dissolved oxygen as a result of the ADV, however, they showed that the droplets, which vaporized during the ADV, are the most important particles that affect the scavenging of the gases.

In real-life applications, fluid characteristics of the blood and droplet concentrations as well as acoustic parameters become relevant to maximize the utility of ADV-generated bubbles and minimize damage. Kang et al. [54] showed that the mean ADV-generated bubble size is smaller in blood plasma than in PBS due to higher viscosity in the former. The acoustic characteristics that showed to affect the size and number of bubbles are mostly pulse duration and pulse repetition frequency (PRF). Further studies may provide validation that the acoustic and flow affect the bubble characteristics in the same way* in vivo*.

The recent studies indicate the significance of the investigation of ultrasound imaging with the aid of PCCA in a realistic microenvironment. There are some studies which show that the confinement elements may affect the oscillating amplitude [55], diameter expansion [56], and signal magnitude [57] of MB negatively. In this regard, Lin et al. [40] demonstrated the effect of the surrounding medium geometry on MB dynamic. They revealed that the lipid-coated PFC droplets exposed to ADV in a microvessel confinement have considerably less acoustic vaporization properties compared to that in a free environment. They claimed that this difference is not limited to tissue attenuation and aberration. A significant decrease in contrast enhancement could also be observed in the microcellulose tube compared to the open environment. The decrease was more significant for B-mode imaging than for pulse inversion imaging. Some of the probable causes of this phenomena include change in surface tension and viscosity of the surrounding medium when it is constrained, and changes in heat transfer and dissipation due to the geometrical confinement.

A summary of recent studies related to droplet fabrication method and their specifications used in bubble activation is reported in Table 1.

## 4. Applications of Acoustic Droplet Vaporization

At low pressures (<10 kPa), the vaporized bubble oscillate linearly, but as the pressure increases (10-100 kPa), and a nonlinear behavior of bubble oscillation is observed. It should be noted that the bubble collapses above a certain pressure amplitude threshold. In this section, the various contemporary and potential future applications are discussed and the requirements on the components are depicted.

### 4.1. Diagnostics

Ultrasound imaging has been used for diagnostics since the 1970's, and it is a technique known for being fast, cheap, and safe compared to computed tomography and magnetic resonance (MR) imaging. This makes it possible to use ultrasound to image flows and dynamic processes in the body, where a high frame rate is required, or to image regions sensitive to radiation. However, the problem with ultrasound is low contrast compared to other modalities, which makes imaging hard in certain regions, where both tissue and liquid are moving, such as myocardium. Enhancing contrast using PCCAs is crucial in order to properly visualize these areas.

The first* in vivo* studies demonstrated that the required acoustic powers for initiation of vaporization are higher than diagnostic ultrasound machines for cases, where nanosized diameters and low frequencies are included [60, 61]. Therefore, subsequent attempts were devoted to fabricating submicron droplets which are stable at a wide range of frequencies, capable of preventing undesirable thermal and cavitation-based bioeffects [18]. The investigations on the bioeffects potential of the nucleated bubbles during the ADV are extended to use them as point targets [62].

One of the major functions of the vaporized droplets is stressed in the drug delivery to increase the efficiency of the tumor therapy and decrease the toxicity. However, they were widely utilized in the early stage diagnosis in the cancer detection [63] and clinical applications due to the high echogenicity, which is beneficial at low concentrations [64]. The subsequent studies revealed the capability of these extravascular contrast agent droplets in the diagnostic imaging techniques [65, 66]. The following studies indicated that it is possible to detect the acoustic characteristics of the vaporized droplets exposed to the low-intensity diagnostic ultrasound shortly after the ADV process with considerably low MI (0.07) for the ultrasound pulse [48].

Diagnostic ultrasound is considered as the crucial step which affects the ADV efficiency toward the successful treatment. One of the major parameters affecting the diagnostic ultrasound scale is the bubble size distribution. Xu et al. [67] utilized a wide beam with low pressure as an acoustic method to determine the bubble size after the ADV process. They illustrated that longer pulse duration and higher PNP increase the populations of big and small generated bubbles during the ADV, respectively. This outcome is considered as the starting point for the development of a distinct pulse for conversion of specific droplets. The related studies showed that it is possible to produce bubbles from typical NDs employing short pulse duration in the diagnostic pressure range [68, 69]. Furthermore, the bubbles generation and their characteristics were studied with the considerations of temperature, initial emulsion size, and frequency at the diagnostic ultrasound level, implying the importance of the generated bubbles specifications in the diagnostic and treatment scales [70]. Although the potential of the nonlinear bubble detection is still in question, it is possible to produce bubbles from the ADV process with uniform size distribution and nonlinear diagnostic acoustic indication with the consideration of the pulse duration, temperature and droplet concentration [44]. In addition, volatility of the perfluorodroplet plays an important role in the diagnostic applications, since lower boiling point induces the droplets to have an earlier vaporization [23].

The generated bubbles from the compressed perfluorodroplets were utilized in diagnostic imaging to reveal the infarct region and diagnosis of the breast cancer [71, 72]. It was shown that it is needed to reveal the physical specifications of the generated bubbles to obtain a desirable ADV efficiency, particularly at the diagnostic stage [73]. Recently, the studies were devoted to the threshold pressure reduction within the diagnostic pressure with the consideration of the boundary conditions limitations, viscosity, and hydrostatic pressure in order to increase the applicability of PCCAs.

In this regards, Rojas et al. [74] employed low-boiling-point PCCAs containing DFB and OFB to utilize their potentials in certain acoustic signatures. The activation pressures of these submicron stable PCCAs are low enough to make them suitable to generate images with high contrast-to-tissue (CTR) ratios. They showed that low threshold pressure of these droplets can be exploited to produce considerable vaporization signals even at short pulse length due to low possibility of bubble coalescence. The authors in their recent work investigated the tube size and pressure effects with the hydrostatic pressure consideration to study the pressure threshold [9]. They found that the pressure threshold raises by increasing the tube size and surrounding viscosity in vivo, which causes increased vaporization in vitro. In an attempt to decrease the pressure threshold, Guo et al. [75] employed an ultrasonic standing wave (USW) while the produced bubbles as a result of ADV were still in the appropriate size range. They found that it is possible to accumulate the dispersed nanodroplets via USW to achieve nanodroplets aggregations. This study is one of the few researches in which USW was utilized to lower the pressure threshold within the diagnostic pressure range.

In a novel method developed by Tremblay-Darveau et al. [76], a pressure-dependent phase lag in the vaporized MB response was observed using amplitude modulation pulse inversion. This phase lag is due to shell softening dynamics after low pressure ultrasound exposure and has no equivalent in tissue, which makes it ideal for imaging. Other techniques, such as 3-D perfusion imaging at low acoustic pressures [10], can significantly improve scan time at high resolutions without risk of heating.

In a recent study, a unique “image-activation-image” protocol was developed and tested that allows both activation and visualization of PCCAs using one and same transducer, both in water bath and to image a rat kidney [42]. The study showed that there are ways to coordinate pulse sequences using only one transducer that allows for both visualization and activation of PCCAs. The activation was accomplished quickly to minimize bubble motion by changing parameters that could be modified fast, e.g., subaperture adjustments and number of cycles in each activation. Initial and final imaging steps were done by pulse inversion imaging over seven different angles and then averaging them. This pulse sequence enables a more common and easy usage of ADV in clinical applications.

### 4.2. Treatment

Although the ADV droplets were primary aimed at applications in diagnostics, many studies show their potential as a part of treatment against various diseases such as cancer [4, 25] and arteriosclerosis [59]. In this section, the goal is to show how ADV could be used in various therapeutic setups. First, medical care with focus on the vascular system is considered. In the following, the possibilities with localized drug delivery using acoustically active droplets are discussed.

#### 4.2.1. Vascular Therapy

Vascular therapy is a broad field, which mostly includes treatment of vascular diseases and various types of tumors, both malign and benign. Depending on the desired outcome, different capsule formulations and acoustic characteristics are preferred. For instance, larger droplets are preferred if vessel occlusion is required since they have a lower vaporization pressure threshold and grow into bigger bubbles, while submicron droplets are more effective when it is important to cause minimal damage to the vessel wall. Since the droplets are not activated unless exposed to acoustic fields, they are safe to use even in microcapillary regions, where the risk of vessel occlusion is high.

It is necessary to know the biological effect of ADV on endothelial cells at various pressure amplitudes for optimization of vascular therapy. This was studied by Seda et al. [3] by subjecting a monolayer of endothelial cells to ADV using a 3.5-MHz transducer and PFP droplets. The results showed that the size of the generated bubble cloud is directly dependent on the pressure amplitude. The larger the pressure amplitude, the bigger the bubble cloud area (BCA). However, increasing pulse length has no effect on BCA, probably because the bubble formation happens during the first few cycles. The cell damage increases with increasing BCA, but it is always within it, which means that the damage is minimized in regions with healthy tissue if they are outside of the focus region. Cell detachment from the surface beneath increases with increasing pressure due to ADV. The combined results showed that ADV can induce cell damage, which is a positive effect when it is requested, e.g., to treat tumor cells in hypoxic regions located far from the blood vessels. On the other hand, it is a negative effect if it is necessary to minimize damage to the vessel walls, e.g., when treating arteriosclerosis. Additionally, a study done by Kang et al. [52] showed that parameters such as droplet concentration, vessel wall stiffness and acoustic characteristics such as PNP, PRF and pulse duration affect the location and depth of lesions from vessel wall erosion. These lesions are particularly pronounced when using high-MI, and are only located on the side proximal to the transducer. All of this should be considered whenever ADV is used in clinical applications.

Cancer tumor cells require high amounts of nutrients and oxygen in order to be able to proliferate. Therefore, they are highly reliant on the presence of blood vessels. By obstructing the feeder vessels that are the main suppliers of the tumor, it is possible to increase the efficiency of other cancer treatments such as chemotherapy and tumor ablation. Samuel et al. [77] showed that vessel occlusion is successful for both capillaries and for the feeder vessel. In capillaries, red blood cell extravasation is observed, when the activation takes place in capillaries, while no vessel wall disruption is observed in the feeder vessel. A probable cause of this phenomenon is the damage done by PFC droplets, when going through inertial cavitation or initial expansion of the PFC gas bubble [59].  Table 2 classifies the recent studies related to vaporization pressure and frequency range corresponding to common cavitation types in acoustic droplet vaporization. As soon as the treatment is over, the bubble successfully dislodged. In a study conducted by Harmon et al. [78], no tumor growth was observed after two weeks of gas embolotherapy, while the tumor size in the control groups had more than doubled. These results together present interesting potential in terms of flexibility and effectiveness in cancer treatment. A summary of droplet fabrication methods and their significances used in vascular therapy are listed in Table 3.

#### 4.2.2. Drug Delivery

**Figure 2:**
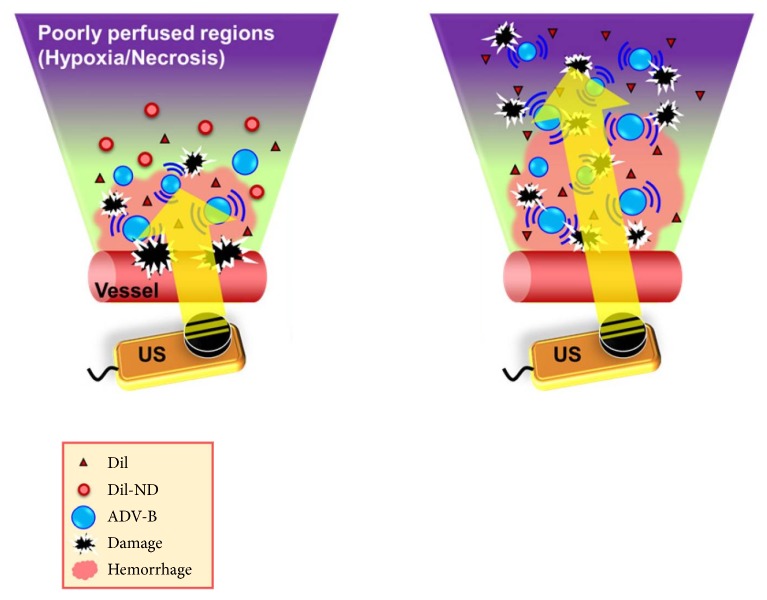
The concept of localized drug delivery to poorly perfused regions. DiI is a red fluorescent dye used to observe the intratumoral distribution. Reprinted (adapted) with premission from [20]. Copyright 2017 American Chemical Society.

Localized drug delivery is a very effective way to maximize efficacy and minimize possible side effects at the same time. This is an especially desired property for cancer treatment, since cytostatic drugs can induce serious complications such as fatigue, difficulty managing everyday tasks at work, and sickness [79]. By using microcapsules in combination with highly focused ultrasound for drug delivery as shown in Figure 2, it is possible to only distribute cytostatic drugs to tumor tissue and not to normal tissues in other regions. Phase-changeable NDs could also be used in combination with liposomes developed for carrying hydrophilic drugs [80]. This is advantageous, since liposomes are more stable than double emulsions.

However, there are several problems with microcapsules that influence their efficiency as drug delivery agents: (1) their limited half-time in the blood stream which reduces their utility and (2) high PNP amplitude required for disruption, which might induce unwanted biological effects such as unnecessary heating and cell lysis. VanOsdol et al. [25] performed a study, where the effects of echogenic low temperature sensitive liposomes (ELTSLs) combined with hyperthermia therapy on drug delivery were explored and compared to echogenic nonthermosensitive liposomes (ENTSLs), nonthermosensitive liposomes (NTSLs), low temperature sensitive liposomes (LTSLs), and free drug distribution. No ADV or its effects on tissue was observed during this study. The different types of liposomes used in this study were similar in size, polydispersity and zeta potential. The results showed that the most effective drug release is achieved, when combining ELTSLs and HIFU, followed by LTSLs, which are combined with HIFU. Previous and current studies determined that the lipid components of NTSLs and ENTSLs are the drug release rate limiting factors. Zonal hyperthermia using HIFU for shorter durations with nanobubble liposome provides larger drug coverage when administered sequentially compared to just irradiating one region. However, if acoustic parameters are optimized, intratumoral penetration can be improved even for NTSLs. This study shows that combining ELTSLs and HIFU can be an effective way to improve drug delivery to a solid tumor.

One way to further increase the efficacy of localized drug delivery is to combine it with anti-vascular therapy, where blood vessels supplying the tumor tissue with nutrients are disrupted, resulting in a much lower risk of tumor regrowth. In a study done by Ho et al. [4], it was demonstrated that combining chemotherapy and anti-vascular therapy by exposing NDs loaded with doxorubicin (DOX) to HIFU would increase tumor cell damage and reduce tumor recurrence compared to separate therapies. The study looked at the separate effects of NDs, free DOX and DOX-NDs both with and without ultrasound exposure at different PNPs and with different cycle numbers. Both short- and long-term effects of exposure to NDs, free DOX and DOX-NDs with and without ultrasound exposure were studied as well. The results showed that the drug extravasation in tumor center is higher when combining DOX-NDs and ultrasound than for any other group due to rupture of small vessels and enhanced permeability and retention (EPR) effect of the drug itself. The short-term effect is the most harmful to the cells; however, long-term exposure is needed for the most prominent chemical effect of the drug. The results showed that it is possible to improve cancer treatment by combining antivascular therapy and chemotherapy. This is also supported by the study done by Ho et al. [24], where they examined how vascular disruption affects drug penetration, when using a combination of ADV and EPR effect. Compared to only EPR and microdroplets exposed to the same acoustic field, NDs outperform in terms of delivering the cytostatics even to hypoxic tumor regions.

Sonoporation is a technique, where ultrasound is used to modify the permeability of cell membrane, which could be used for more efficient drug and gene delivery. Fix et al. [19] produced capsules with a core made out of PFCs with a boiling point below body temperature and studied if their vaporization affected sonoporation potential and cell viability of human pancreatic adenocarcinoma cells. The results showed that vaporization threshold is lower for OFP-filled PCCAs than for PFP-filled PCCAs and that it is possible to control the severity of cellular bioeffects by modifying pulse length. More studies must be done to understand how to optimize sonoporation.

Soto et al. [11] recently developed a wearable transdermal patch, which consists of a drug reservoir containing hundreds of micropores loaded with lidocaine. The drug is released upon ultrasound exposure, which enables faster and deeper penetration on the skin compared to non-ADV-generating ultrasound exposure and therefore a more efficient drug release. Development of this technique and further decrease in drug release rate might provide improvement in pain relief treatment.

Other diseases than cancer might also benefit from using ADV. Many drugs currently developed for various neurological diseases are large proteins, which are blocked by the blood-brain barrier (BBB) and therefore do not reach the brain. Lipsman et al. [82] reported that the blood-brain barrier is successfully opened in a safe, reversible, and repeatable way when MR-guided focused ultrasound was used in combination with acoustically active MBs in five patients with Alzheimer's in a safety trial. No serious clinical or radiographic adverse events were reported, suggesting that acoustically active NDs may have potential as drug carriers for these type of patients in the future. Wu et al. [83] used OFP and DFB droplets separately to deliver 40 kilodalton dextran (size relevant to proteins) to mice brain and compared the results to MBs. It is shown that substantially more OFP NDs vaporize at low acoustic pressures (300-450 kPa) in the* in vitro* study compared to DFB droplets at higher pressures (750-900 kPa). This trend is also reported in other studies [84]. In the* in vivo* study, successful delivery of dextran is achieved, while DFB droplets produce tissue damage due to inertial cavitation. This suggests that OFP NDs could have the potential to be used for drug delivery in the brain. To ensure that no permanent harm is done to potential patients, further studies regarding safety of microcapsules and their effect on the vascular system of the brain at various acoustic parameters are necessary.  Table 4 reports the recent studies within drug delivery with considering droplet fabrication methods.

#### 4.2.3. Theranostics

Theranostics is a new field in medical application which combines diagnostics and treatment, allowing faster and more flexible treatments of various pathologies. Due to monitoring before and after the droplet collapse, it is possible to deliver drugs more precisely. Theranostics using phase-changeable agents is therefore suitable for tumor treatment, where it is important to reach the entire volume of the tumor, including poorly perfused regions. In a study performed by Ho et al. [20], NDs were applied to investigate the behaviors of intertissue ADV-generated bubbble (ADV-B) formation, movement, and cavitation by intravital imaging. The interaction between cells and ADV-B movement was evaluated through* in vitro* cellular experiments to simulate the possible bioeffects. Fluorescent dyes were used to observe intratumoral distribution of NDs and ADV-Bs by histological assessment and to study the bioeffects on tumor cells during ADV and ADV-B movement. It could also be seen that the formation and movement of the ADV-Bs depend on PNP amplitude: new intertissue ADV-Bs were formed only at 10 MPa and ADV-Bs moved at both 5 MPa and 10 MPa but moved more at higher PNPs. The results also showed that ADV-Bs could diffuse into poorly perfused tumor regions and cause significant cell damage and cell death when exposed to ultrasound. In the future, the process of intertissue ADV-B movement pushing ADV-Bs together to create larger ADV-Bs or bubble clouds can be studied.

Cancer cells proliferate much more than healthy cells and might therefore have significant overexpression of certain receptors. This can be used in theranostics to achieve an even more precise drug delivery when treating various types of cancer. Folate is required for replication of deoxyribonucleic acid (DNA) in most human cancer cells, and there is an overexpression of folate receptors (FR) on their membranes. Chen et al. [12] tested the feasibility of folate-conjugated drug-loaded acoustic NDs (FA-CPT-NDs) and compared their performance to drug-loaded NDs without conjugated folate, blank NDs, and free drug. The tests were performed* in vitro* and in a mouse xenograft model using one FR-positive (KB, human epidermal carcinoma) and one FR-negative (HT 1080, human fibrosarcoma) cell lines. The results showed that FA-CPT-NDs successfully went through vaporization and had a significantly higher cell killing effect of KB tumor than HT 1080 tumor, while not causing severe damage in surrounding tissue. It shows that FA-CPT-NDs were more effective in targeting only the tumor compared to the control particles. A slight cell death was observable when FA-CPT-NDs were not exposed to ultrasound, which was probably due to leakage of drug from the capsule. Similar results were obtained in a study performed by Marshalek et al. [86], where FR-positive breast cancer cells were used as an* in vitro* model. This technique showed promising results and could be further improved by optimizing the drug dosage and ND concentration in blood, as well as acoustic characteristics.

A different approach that has gained interest in recent years is to let adipose-derived stem cells loaded with acoustically active NDs migrate to tumor intertissue, where they are exposed to ultrasound. Ho et al. [13] showed that camptothecin-loaded NDs successfully went through intracellular ADV, increasing in size by a factor of 12 and disrupting the carrier cell. The camptothecin-loaded NDs were designed to be able to fuse together to form larger droplets, which lowered the vaporization pressure threshold and enabled a more efficient drug release and contrast-enhanced ultrasound imaging.

**Figure 3:**
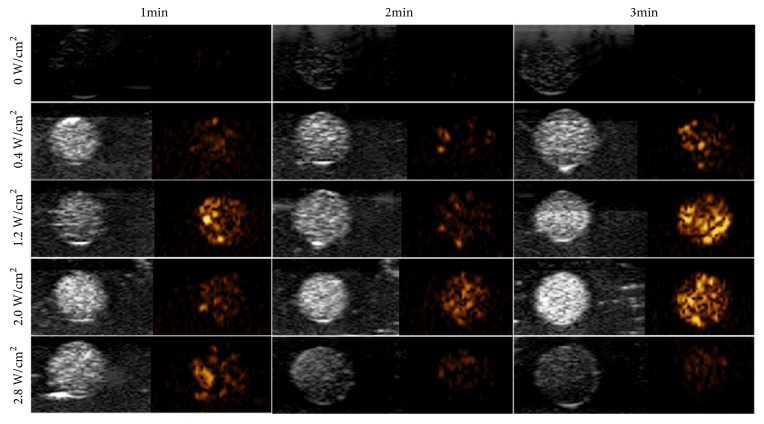
Ultrasound B-mode (left) and contrast-enhanced ultrasound mode (right) images of peptide-functionalized drug-loaded nanoparticles at different time points of various LIFU settings. At high enough power, the nanoparticles exploded, which could be seen as a decrease in signal intensity at 2.8 W/cm^2^. Reprinted (adapted) with premission from [81]. Copyright 2018 American Chemical Society.

Recently, theranostics utilizing low intensity focused ultrasound (LIFU) has gained interest due to reduced risk of damage to healthy tissue during treatment compared to HIFU. Zhu et al. [81] showed that, with LIFU assistance, peptide-functionalized phase-changeable NDs were able to successfully target human breast adenocarcinoma cells in a xenograft model and release the drug 10-Hydroxycamptothecin (10-HCPT), thus reducing the tumor size significantly compared to non-targeted NDs. It was also possible to image the droplets before activation using B-mode and contrast-enhanced ultrasound imaging, which can be seen in Figure 3. These results are similar to those obtained by Wang et al. [87]. They developed a biocompatible cetuximab-conjugated phase-change capsules (H-CPN) that resulted in a cell viability for C643 cells of 23.5% ± 1.29% after 3 minutes of LIFU exposure, in comparison with a cell viability of 31.09% ± 1.57% for free 10-HCPT. H-CPN showed also considerable contrast enhancement during B-mode and contrast-enhansed ultrasound (CEUS) mode imaging, as well as improved therapeutic effect of the drug. LIFU has also shown to be effective for phase-transition of NDs with different types of shells for customized drug release rate [88]. More studies are required for optimization of this technique for various tumor treatments and to assess its potential long-term risks.

#### 4.2.4. Thermal Therapy

MBs exposed to HIFU have been used for tumor ablation in both benign and malign tissue [95]. The disadvantages of using MBs are their short circulation time* in vivo* and their size, which limits them to the vascular system. A feasible solution would be to instead use acoustically active NDs [21]. The droplets used by Moyer et al. were comprised of a 1:1 ratio of DFB and PFP so that the bubbles required a lower activation pressure threshold and therefore cause less damage to normal tissue. A rat liver was used as an* in vivo* model. The results showed that while MBs had their thermal peak at the skin surface, the peak for NDs was close to the HIFU focal point and therefore caused less thermal damage to healthy tissue. A limitation of the study was the lack of substantial depths which would result in attenuation effects; however, this is nevertheless interesting results and further studies could highlight the advantages of using NDs for ablation.

One disadvantage currently seen with HIFU thermal therapy is long treatment times. This is especially problematic if the region to be treated are large. Kripfgans et al. [15] have proposed a new activation method that allows for controlled, fast, and effective lesion ablation using ADV-generated bubbles. MR and optical measurements were done to visualize the ADV process and the ablation. Egg-white based phantoms were used since the proteins in them denaturate and become opaque if exposed to heating of >50°C for more than 5 seconds. The HIFU heating was performed following a spiral pattern, which reflected and preserved some of the heating in between the “walls” of the spiral, causing ADV-generated bubbles in close regions. By using this approach, a 15-time increase in volume was accomplished while only increasing treatment time by 37%.

A current limitation is that this technique is limited to superficial tumors due to attenuation of ultrasound at higher depths. A novel method developed by Xin et al. [96] utilizes LIFU and a pulse wave-continuous wave which formed larger bubble clouds than if only a continuous wave was used. Droplet concentration and vaporization pressure of the pulse wave also influenced the volume of the bubble cloud and enabled production of lesions of various sizes and shapes. More research is needed to understand the underlying mechanisms, which could in turn help optimizing the treatment.

#### 4.2.5. Histotripsy

Histotripsy is a nonthermal noninvasive method for treating tumors by mechanical ablation caused by inertial cavitation. The method is based on applying short, focused, high-pressure ultrasound pulses to generate a cavitation bubble cloud inside the treated tissue. The frequencies used in current clinically approved applications are typically below 1 MHz to further enhance the cavitation, which is inversely proportional to the frequency [97]. Although this technique has shown great potential for treating pathologies such as kidney stones [98] and liver cancer [99], its efficacy is limited to situations in which a single target can be accurately visualized and linearized prior to treatment [100]. Combined with the difficulty to image the region of interest, there is a risk of unwanted bioeffects in surrounding healthy tissue due to the high pressures (>28 MPa) required for successful procedure.

Nanodroplet-mediated histotripsy (NMH) is a novel technique developed to overcome the drawbacks of conventional histotripsy. The cavitation threshold of nanodroplet-mediated histotripsy is determined by the incident PNP [101], the frequency [102], and the elasticity of the surrounding medium [103]. For all studies involved, the cavitation threshold was significantly lower in NMH compared to conventional histotripsy [16, 102, 104], which would considerably reduce the risk of damaging healthy tissue outside the region of interest.

NDs used for histotripsy are usually made of a triblock copolymer shell for increased droplet stability [105]. However, there are a few issues associated with currently developed NDs. First, the synthesis is complex and requires high competence in polymer chemistry. Second, the size of NDs could be further reduced for better accumulation in tumor tissue. Third, the concentration of PFP is vital for the cavitation threshold. Rehman et al. [16] have recently developed a novel type of nanocone (NC) made out of methylated *β*-cyclodextrin and filled with PFP. The NCs have a size smaller than 50 nm, which make them suitable for diffusion into the tumor intertissue. They are also made by mixing two commercially available and FDA approved substances in a simple manner [16]. Khirallah et al. [17] showed that the cavitation threshold decreases, when the concentration of these NC increases. This is an interesting result and should be further investigated to counterbalance potential safety risks for the patient. A lower frequency is preferable for NMH, since it not only decreases the cavitation threshold but also increases bubble expansion and increases the size of the focal region [102], therefore enabling faster treatment of larger tumors.

## 5. Recent Developments in the Field

The recent studies were devoted to solve the substantial problems regarding the ultrasound visualization when ADV is utilized. Liu et al. [89] fabricated a novel class of folate-targeted PFP NDs which were considerably stable and small. They showed that the fabricated droplets in combination with the LIFU increases the efficiency of the ultrasound molecular probes in terms of contrast enhancement. The related investigations reported a successful early detection of ovarian cancer with the aid of stable folate-targeted NDs in vivo and in vitro [90]. Moreover, condensation and cosolvent dissolution were employed to overcome the ADV restrictions in terms of the generated bubbles size, monodispersity, and controllability and to reduce the size of cosolvent-infused PFC bubbles [91]. In addition, it was addressed in the literature that the new class of the NDs has the capability of providing multimodel imaging [92] and its effectiveness in the gene expression was reported as a result of the localized drug releasement [106]. Therefore, it is possible for the small particles to penetrate in the tumor tissue and convert to destructive bubbles as a result of the ultrasound activation [85]. The aforementioned studies presented a substantial progress in the treatment and imaging of cancer with the use of ultrasound contrast agents.

The noninvasive nature of the ADV has a great advantage in cancer treatment. This fact depicted its capability in the gas embolization with the aid of focused ultrasound and excluding the interartial catheter in the hepatocellular carcinoma treatment [78]. The proposed method as an alternative for the current embolization method eliminates the invasive treatment limitations when catheter is utilized [107]. The recent studies illustrated that the NDs emulsion used in the ADV is not only influential due to its noninvasive nature, but also it is possible to harvest the energy absorption and to control the thermal dissipation particularly in highly vascular organs during the cancer treatment [93]. Being able to monitor the heat dissipation helps one to detect the nucleation and cavitation bubbles generation, which is essential in guiding the vaporized droplets to the target properly [108]. Besides the utilization of the ADV in the cancer and tumor treatment and its strong features as an alternative to the invasive current methods, the ADV process itself was developed and some studies reported in the literature went through the limitation arose from the instant vaporization. In this regards, Zhang et al. [94] indicated that it is possible to receive a continuous respond from the encapsulated NDs emulsion during radiofrequency (RF) ablation and avoid the long irradiation period and high amount of power dissipated during the radiofrequency [109]. Therefore, it is possible to improve the imaging quality while the RF ablation is enhanced via lowering the output power with the aid of metal-based nanoparticles used in the encapsulated droplets.

Although the recent studies demonstrated broad applicability of the ADV process and its substantial effects on the transformative of current techniques in cancer and tumor therapy, there are still rooms in identifying the characterization of droplets from their size to the utilization particularly in the imaging quality and reticuloendothelial system to avoid of clogging. The recent developments in the field of ADV are reported in Table 5.

## 6. Summary

Acoustic droplet vaporization (ADV), a physical process where a capsule with a superheated liquid core goes through phase transition after ultrasound exposure, has in recent years gained interest in biomedical applications. The dynamic behavior of bubbles produced after ADV depends on acoustic pressure, frequency, shell and core material properties, droplet concentration, and the geometry of the surroundings. Today, there are many different manufacturing techniques available for production of acoustically active capsules that utilize sonication, microfluidics, or condensation of gas cores to the liquid state. These acoustically active capsules of various sizes, as well as shell and core materials, have been developed and adapted to a wide range of medical applications such as diagnostic imaging, drug delivery, theranostics, thermal ablation, histotripsy, and vascular therapy. The optimal design of microcapsules depends on the intended usage; for vascular therapy, larger and more stable droplets are required, while it is more advantageous to use small (<500 nm) droplets for cancer therapy that can leak from the blood vessels into intermediary tissue. Recent work has focused on techniques that utilized LIFU, because of its lower risk of mechanical damage outside the regions of interest, development of targeted drug-loaded capsules for precise drug delivery, and treatment of neurological pathologies. For successful translation into the clinic, it is necessary to verify that phase-changeable droplets are safe to use in humans and what the exact requirements on the acoustic field should be. It is crucial to evaluate the risks, which could be assessed in future works.

## Figures and Tables

**Table 1:** Droplet fabrication method, significances and specifications used in bubble activation.

Study	Core material	Shell	Fabrication method	Size before ADV	Size after ADV	Significance	Future potential
Li et al. [37]	Perfluoropentane	Albumin	Mixing Saline containing PBS albumin and perfluoropentane	Various sizes of micrometer scale	36 *μ*m	A model that describes the location of initial nucleation site in liquid PFC droplets depending on droplet size and acoustic characteristics	The initial nucleation site always occurs within the droplet and not at the bubble-surface interface

Miles et al. [38]	Perfluoropentane	Lipids	NA	3-30 *μ*m	NA	Possible to predict ADV initiation pressure threshold by combining classical nucleation theory with superharmonic focusing	Customize droplet size and/or pressure in advance depending on the application

Strohm et al. [22]	Perfluorohexane	Fluorosurfactant (Zonyl FSO)	Coarse emulsification by vortexing, followed by membrane emulsification	2.45-13.6 *μ*m	NA	Only the physical properties of the PFH liquid determines the droplet photoacoustic spectrum features	Proof of concept

Doinikov et al. [39]	Octafluoropropane and decafluorobutane	1,2-distearyl-sn-glycero-3-phosphocholine, DSPE-PEG2000	Condensation	2.2-6.7 *μ*m	Up to 40 *μ*m	A new model that describes the dynamic behavior of highly volatile perfluorocarbon emulsions, which was in good agreement with experimental results	Predict the behavior of highly volatile perfluorocarbon emulsions at various acoustic parameter values

Lin et al. [40]	Decafluorobutane	DPPC, 16:0 PEG2000 PE	Condensation	243 nm	1.22 *μ*m	PCCAs in microchannel will have a lower vaporization efficiency than PCCAs in free environment at the same acoustic conditions	Higher validity in conclusions drawn from images with microvasculature

Sheeran et al. [41]	Perfluoropropane or perfluorobutane	1,2-distearoyl-sn-glycero-3-phosphocholine, DSPE-PEG2000	Condensation	192 nm	1.2 *μ*m (DFB), 1.6 *μ*m (OFP)	Unique acoustic signals are generated by vaporizing phase-change droplets that can be detected and isolated from other acoustic sources	Combine different imaging modalities for optimal diagnostics

Puett et al. [42]	Decafluorobutane or octafluoropropane	DSPC, DSPE-PEG2000	Condensation	240 nm	NA	A new pulse sequence design that enables fast image-activation-image process	Fast and easy study of ADV activated droplets in research

Li et al. [43] (method developed by Paul S. Sheeran et al. [18])	Decafluorobutane	Lipids	Condensation	230-350 nm	5-6.5 *μ*m	Linear relationship between droplet concentration and activation; at room temperature, the bubbles were fewer and larger compared to body temperature	Control of droplet concentrations in applications that use inertial cavitation

Reznik et al. [44]	Perfluoropentane	Fluorosurfactant (Zonyl FSO)	Tip-sinocation approach	400 nm	500 nm - 5 *μ*m	The coating material highly influence the acoustic behavior of vaporized PFC droplets	Fluorosurfactant-coated droplets has potential as contrast agents in ultrasound imaging

**Table 2:** Vaporization pressure and frequency range corresponding to common cavitation types in acoustic droplet vaporization.

Cavitation type	Vaporization frequency	Vaporization pressure	Imaging frequency	Application	Study
Stable	5 MHz	3.5 MPa	2.5 MHz (acoustic characterization)	Bubble activation	Reznik et al. [48]
	5 MHz	2.5-4 MPa	NA	Capsule design; Diagnostics	Martz et al. [27]
	7.5 MHz	Depends on droplet size	7.5 MHz	Bubble activation	Li et al. [37]
	7.5 MHz	Depends on droplet size	NA	Bubble activation	Miles et al. [38]
	7.5 MHz	1.6 MPa	NA	DIagnostics; Theranostics	Jin et al. [7]
	8 MHz	500 kPa	4.5 MHz	Bubble activation; Diagnostics	Doinikov et al. [39]
	8 MHz	3.39 MPa	4.5 MHz	Diagnostics; Drug delivery	Lin et al. [8]
	8 MHz	4.81 MPa	1 MHz (passive recording)	Bubble activation	Lin et al. [40]
	8 MHz	2.5 MPa (for OFP), 3.7-4.2 MPa (for DFB)	7 MHz; 14MHz	Diagnostics	Lin et al. [58]
	8 MHz	3.1 MPa	NA	Diagnostics	Paul S. Sheeran et al. [33]
	9 MHz	3.7-8.7 MPa (for DFB), 3.1-7.4 MPa (for OFP)	4.5 MHz	Bubble activation; Diagnostics	Puett et al. [42]

Inertial	1 MHz	4.41 MPa	NA	Drug delivery	VanOsdol et al. [25]
	1.5 MHz	90 W (for vaporization), 5.2-38.5 W (for inertial cavitation)	NA	Thermal therapy	Kopechek et al. [14]
	1.14 MHz	4 MPa	NA	Thermal therapy	Moyer et al. [21]
	2 MHz	NA	2 MHz	Drug delivery	Duncanson et al. [30]
	2.25 MHz	12 V	NA	Drug delivery	Soto et al. [11]
	1.44 MHz	7.4 MPa	NA	Thermal therapy	Kripfgans et al. [15]
	2 MHz	7-10 MPa	5 MHz (passive cavitation detection), 2.25 MHz (attenuation)	Vascular therapy	Kang et al. [52]
	1.513 MHz	5.8 MPa (in vitro), 88 W (in vivo)	NA	Vascular therapy	Pajek et al. [59]

**Table 3:** Droplet fabrication method, significances, and specifications used in vascular therapy.

Study	Core material	Shell	Fabrication method	Size before ADV	Size after ADV	Significance	Future potential
Samuel et al. [77]	Perfluoropentane	Albumin	Based on mixing saline containing bovine serum albumin and perfluoropentane	2 *μ*m	76 × 36 *μ*m (in capillaries), 25 × 11 *μ*m (in feeder vessel)	Vaporizing in the capillaries causes vessel damage and subsequent leakage of blood cells, but no vessel damage was seen for the feeder vessel	Further improve and optimize gas embolotherapy using ADV-generated PFC bubbles

Kang et al. [52]	Perfluoropentane	DPPC, DSPE-PEG2000, cholesterol	Sonication of a vial containing shell mixture and perfluoropentane	1.3 *μ*m	Up to 8 *μ*m	Possible to predict the mechanical damage severity as a function of droplet vaporization, PNP, phantom stiffness, pulse duration and PRF	Improved control of damage done by ADV in medical applications

Seda et al. [3]	Perfluoropentane	Albumin	Sonication of a vial containing shell mixture and perfluoropentane	1.6 *μ*m	NA	Viability of endothelial cells is lower at the ADV bubble site than at other regions	Control of damage during gas embolotherapy

Pajek et al. [59]	Perfluoropentane	Fluorosurfactant (Zonyl FSO)	Sonication of a vial containing shell mixture and perfluoropentane	136-152 nm (in vitro), 156-207 nm (in vivo)	NA	A lower pressure amplitude is sufficient to induce inertial cavitation if droplets with perfluorocarbon is present in sonothrombolysis	Reduced risk of thermal damage in healthy tissue while efficient sonothrombolysis

**Table 4:** Droplet fabrication method, significances and specifications used in drug delivery.

Study	Core material	Shell	Fabrication method	Size before ADV	Size after ADV	Significance	Future potential
Ho et al. [4]	Perfluoropentane	DPPC, DSPG, DSPE-PEG5000	Condensation	214 nm	100% of vessels with diameters below 10 *μ*m were disrupted	Drug-loaded capsules are used to disrupt blood vessels and deliver drugs locally to a tumor	Improved tumor treatment by obstructing blood flow and causing direct damage

VanOsdol et al. [25]	Perfluoropentane	DPPC, MSPC, DSPE-mPEG2000; NTSL: DPPC, cholesterol, DSPE-mPEG2000	Extrusion method for synthesizing lipid shell and pH-gradient loading protocol for droplet preparaion	170-190 nm	NA	Echogenic low temperature sensitive liposomes are effective for drug delivery when paired with sequential HIFU heating for short duration time	Improved treatment of difficult cancers, such as lvier and colon

Rapoport et al. [85]	Perfluoropentane	Block polymer stabilized micelles	A solvent exchange echnique for micellar solution and transferring PFP to the sonicated shell	Three peaks: 19.3-29.3 nm, 114.7-188.9 nm, 592.6-718.4 nm	Up to 10 *μ*m	Drug-loaded emulsions combined with HIFU can be effective when treating ovarian, breat and pancreatic cancer	Need to study cell resistance, since second treatment was not at all as efficient as the first one

Fix et al. [19]	Perfluoropropane	DSPC, DSPE-PEG2000	Condensation	140 nm	1-10 *μ*m	Low-boiling PCCAs induce cellular sonoporation, and efficiency of sonoporation increase with increasing PNP and increasing pulse length	Improved drug or gene delivery

Soto et al. [11]	Perfluorocarbon	DSPC, mPEG-DSPE	Evaporation of the mixed nanoemulsion with the use of an ultrasound probe generator	NA	NA	The patch loaded with lidocaine and PFC emulsions and exposed to US increased the payload skin penetration compared to passive diffusion	New way to deliver local anesthetics over a long time

Ho et al. [24]	Perfluoropentane	DPPC, DSPG, DSPE-PEG2000	Condensation	1.1 *μ*m (MDs), 124 nm (DLs), 357 nm (NDs)	10-15 *μ*m	Vascular disruption induced by ADV with multiple US sonications improves drug penetration more than the EPR effect	Better drug delivery optimization

**Table 5:** Summary of studies on attempts for improvement in acoustic droplet vaporization.

Purpose	Advantage in relation to ADV	Outcome	Application	Droplet core and specification	Study
Fabrication of novel ultrasound molecular probe	Use of low-intensity focused US (LIFU) sonication for the regular ultrasound visualization	Notable ultrasound contrast enhancement in the tumor region	Tumor molecular imaging	Folate-targeted; Perfluoropentane	Liu et al. [89]

Synthesize a targeted computed tomography (CT) perfluorooctylbromide nanoparticle for the early detection of ovarian cancer	Successful targeted drug delivery	High efficiency in terms of targeting and long circulation time of the synthesized droplets	Detection of ovarian cancer	Folate-receptor-targeting; Perfluorooctylbromide	Liu et al. [90]

Fabrication of droplets with size control using microfluidics	Viability of the droplets for the in situ production of ultrasound contrast agents as a echogenic bubbles	Fabrication of droplets have a size of 24 times smaller than the precursor bubbles	Cancer imaging and therapy	Monodisperse; Cosolvent-incorporated; Perfluorocarbon	Seo et al. [91]

Fabrication of a multifunctional targeted poly(lactic-co-glycolic acid) (PLGA) nanobubbles for tumor imaging and HIFU ablation	The combination of HIFU ablation and chemotherapy with the aid of the developed NBs suits well with several therapeutic and diagnostic treatments	The change in the acoustic environment leads to improve the efficiency of HIFU ablation when the fabricated NBs are using	Tumor imaging; Anti-cancer drug carrier; Synergistic agent for enhancing therapeutic efficiency	Methotrexate (MTX)-loaded NBs filled with perfluorocarbon	Zhang et al. [92]

Targeted gas embolotherapy with the aid of ADV	The feasibility of occluding vessels with in the embolotherapy with the use of ADV	The successful utilization of gas embolotherapy as an noninvasive method for the treatment of unresectable hepatocellular carcinoma	Hepatocellular carcinoma	Perfluoropentane	Harmon et al. [78]

Locally enhance energy absorption and heating in the focused ultrasound	Suitability of the nanoemulsion in the thermal FUS therapies	Enhancement of acoustic cavitation and temperature rise in an in vitro study with the use of phase-shift nanoemulsions (PSNE)	Thermal focused ultrasound therapy	Perfluoropentane	Crake et al. [93]

To lower the power output and radiation time during radiofrequency (RF) ablation	Using the versatility of radiofrequency solidoid vaporization to compensate drawbacks and limitations of ADV and optical droplet vaporization (ODV)	Employment of continuous cavitation to enhance the RF ablation	Cancer theranostics	Perfluorocarbon-encapsulated theranostic agents	Zhang et al. [94]

## References

[1] Gramiak R., Shah P. M. (1968). Echocardiography of the aortic root. *Investigative Radiology*.

[2] Ziskin M. C., Bonakdarpour A., Weinstein D. P., Lynch P. R. (1972). Contrast agents for diagnostic ultrasound. *Investigative Radiology*.

[3] Seda R., Li D. S., Fowlkes J. B., Bull J. L. (2015). Characterization of bioeffects on endothelial cells under acoustic droplet vaporization. *Ultrasound in Medicine & Biology*.

[4] Ho Y.-J., Yeh C.-K. (2017). Concurrent anti-vascular therapy and chemotherapy in solid tumors using drug-loaded acoustic nanodroplet vaporization. *Acta Biomaterialia*.

[5] Rapoport N. Y., Kennedy A. M., Shea J. E., Scaife C. L., Nam K. (2009). Controlled and targeted tumor chemotherapy by ultrasound-activated nanoemulsions/microbubbles. *Journal of Controlled Release*.

[6] Zhu M., Jiang L., Fabiilli M. L., Zhang A., Fowlkes J. B., Xu L. X. (2013). Treatment of murine tumors using acoustic droplet vaporization-enhanced high intensity focused ultrasound. *Physics in Medicine and Biology*.

[7] Jin Q., Lin C., Kang S. (2017). Superhydrophobic silica nanoparticles as ultrasound contrast agents. *Ultrasonics Sonochemistry*.

[8] Lin S., Shah A., Hernández-Gil J. (2017). Optically and acoustically triggerable sub-micron phase-change contrast agents for enhanced photoacoustic and ultrasound imaging. *Photoacoustics*.

[9] Juan Rojas D., Paul Dayton A. (2019). Vaporization detection imaging: A technique for imaging low-boiling-point phase-change contrast agents with a high depth of penetration and contrast-to-tissue ratio. *Ultrasound in Medicine Biology*.

[10] Tremblay-Darveau C., Sheeran P. S., Vu C. K., Williams R., Bruce M., Burns P. N. (2018). 3D perfusion imaging using principal curvature detection rendering. *IEEE Transactions on Ultrasonics, Ferroelectrics and Frequency Control*.

[11] Soto F., Jeerapan I., Silva-López C. (2018). Noninvasive transdermal delivery system of lidocaine using an acoustic droplet-vaporization based wearable patch. *Small*.

[12] Chen W., Kang S., Lin J., Wang C., Chen R., Yeh C. (2015). Targeted tumor theranostics using folate-conjugated and camptothecin-loaded acoustic nanodroplets in a mouse xenograft model. *Biomaterials*.

[13] Ho Y., Chiang Y., Kang S., Fan C., Yeh C. (2018). Camptothecin-loaded fusogenic nanodroplets as ultrasound theranostic agent in stem cell-mediated drug-delivery system. *Journal of Controlled Release*.

[14] Kopechek J. A., Park E., Mei C.-S., McDannold N. J., Porter T. M. (2013). Accumulation of phase-shift nanoemulsions to enhance mr-guided ultrasound-mediated tumor ablation in vivo. *Journal of Healthcare Engineering*.

[15] Kripfgans O. D., Zhang M., Fabiilli M. L. (2014). Acceleration of ultrasound thermal therapy by patterned acoustic droplet vaporization. *The Journal of the Acoustical Society of America*.

[16] Rehman T. U., Khirallah J., Demirel E., Howell J., Vlaisavljevich E., Yuksel Durmaz Y. (2019). Development of acoustically active nanocones using the host–guest interaction as a new histotripsy agent. *ACS Omega*.

[17] Khirallah J., Schmieley R., Demirel E. Nanoparticle-mediated histotripsy (NMH) using perfluorohexane "nanocones". *Physics in Medicine & Biology*.

[18] Sheeran P. S., Luois S., Dayton P. A., Matsunaga T. O. (2011). Formulation and acoustic studies of a new phase-shift agent for diagnostic and therapeutic ultrasound. *Langmuir*.

[19] Fix S. M., Novell A., Yun Y., Dayton P. A., Arena C. B. (2017). An evaluation of the sonoporation potential of low-boiling point phase-change ultrasound contrast agents in vitro. *Journal of Therapeutic Ultrasound*.

[20] Ho Y., Yeh C. K. (2017). Theranostic performance of acoustic nanodroplet vaporization-generated bubbles in tumor intertissue. *Theranostics*.

[21] Moyer L. C., Timbie K. F., Sheeran P. S., Price R. J., Miller G. W., Dayton P. A. (2015). High-intensity focused ultrasound ablation enhancement in vivo via phase-shift nanodroplets compared to microbubbles. *Journal of Therapeutic Ultrasound*.

[22] Strohm E. M., Gorelikov I., Matsuura N., Kolios M. C. (2012). Acoustic and photoacoustic characterization of micron-sized perfluorocarbon emulsions. *Journal of Biomedical Optics*.

[23] Sheeran P. S., Luois S. H., Mullin L. B., Matsunaga T. O., Dayton P. A. (2012). Design of ultrasonically-activatable nanoparticles using low boiling point perfluorocarbons. *Biomaterials*.

[24] Ho Y. H., Chang Y. C., Yeh C. K. (2016). Improving nanoparticle penetration in tumors by vascular disruption with acoustic droplet vaporization. *Theranostics*.

[25] VanOsdol J., Ektate K., Ramasamy S. (2017). Sequential HIFU heating and nanobubble encapsulation provide efficient drug penetration from stealth and temperature sensitive liposomes in colon cancer. *Journal of Controlled Release*.

[26] Lee J. Y., Carugo D., Crake C. (2015). Nanoparticle-loaded protein-polymer nanodroplets for improved stability and conversion efficiency in ultrasound imaging and drug delivery. *Advanced Materials*.

[27] Martz T. D., Sheeran P. S., Bardin D., Lee A. P., Dayton P. A. (2011). Precision manufacture of phase-change perfluorocarbon droplets using microfluidics. *Ultrasound in Medicine & Biology*.

[28] Martz T. D., Bardin D., Sheeran P. S., Lee A. P., Dayton P. A. (2012). Microfluidic generation of acoustically active nanodroplets. *Small*.

[29] Teston E., Hingot V., Faugeras V. (2018). A versatile and robust microfluidic device for capillary-sized simple or multiple emulsions production. *Biomedical Microdevices*.

[30] Duncanson W. J., Arriaga L. R., Ung W. L., Kopechek J. A., Porter T. M., Weitz D. A. (2014). Microfluidic fabrication of perfluorohexane-shelled double emulsions for controlled loading and acoustic-triggered release of hydrophilic agents. *Langmuir*.

[31] Fabiilli M. L., Lee J. A., Kripfgans O. D., Carson P. L., Fowlkes J. B. (2010). Delivery of water-soluble drugs using acoustically triggered perfluorocarbon double emulsions. *Pharmaceutical Research*.

[32] Capece S., Chiessi E., Cavalli R., Giustetto P., Grishenkov D., Paradossi G. (2013). A general strategy for obtaining biodegradable polymer shelled microbubbles as theranostic devices. *Chemical Communications*.

[33] Sheeran P. S., Streeter J. E., Mullin L. B., Matsunaga T. O., Dayton P. A. (2013). Toward ultrasound molecular imaging with phase-change contrast agents: an in vitro proof of principle. *Ultrasound in Medicine & Biology*.

[34] Juan Rojas D., Paul Dayton A. (2019). In vivo molecular imaging using low-boiling-point phase-change contrast agents: a proof of concept study. *Ultrasound in Medicine & Biology*.

[35] Chen P.-K., Lai N.-C., Ho C.-H., Hu Y.-W., Lee J.-F., Yang C.-M. (2013). New synthesis of MCM-48 nanospheres and facile replication to mesoporous platinum nanospheres as highly active electrocatalysts for the oxygen reduction reaction. *Chemistry of Materials*.

[36] Yildirim A., Dennis S., Shambojit R. (2018). Nanoparticle-mediated acoustic cavitation enables high intensity focused ultrasound ablation without tissue heating. *ACS Applied Materials & Interfaces*.

[37] Li D. S., Kripfgans O. D., Fabiilli M. L., Brian Fowlkes J., Bull J. L. (2014). Initial nucleation site formation due to acoustic droplet vaporization. *Applied Physics Letters*.

[38] Miles C. J., Doering C. R., Kripfgans O. D. (2016). Nucleation pressure threshold in acoustic droplet vaporization. *Journal of Applied Physics*.

[39] Doinikov A. A., Sheeran P. S., Bouakaz A., Dayton P. A. (2014). Vaporization dynamics of volatile perfluorocarbon droplets: a theoretical model and in vitro validation. *Medical Physics*.

[40] Lin S., Zhang G., Leow C. H., Tang M.-X. (2017). Effects of microchannel confinement on acoustic vaporisation of ultrasound phase change contrast agents. *Physics in Medicine and Biology*.

[41] Sheeran P. S., Matsunaga T. O., Dayton P. A. (2014). Phase change events of volatile liquid perfluorocarbon contrast agents produce unique acoustic signatures. *Physics in Medicine and Biology*.

[42] Puett C., Sheeran P. S., Rojas J. D., Dayton P. A. (2014). Pulse sequences for uniform perfluorocarbon droplet vaporization and ultrasound imaging. *Ultrasonics*.

[43] Li S., Lin S., Cheng Y., Matsunaga T. O., Eckersley R. J., Tang M.-X. (2015). Quantifying activation of perfluorocarbon-based phase-change contrast agents using simultaneous acoustic and optical observation. *Ultrasound in Medicine & Biology*.

[44] Reznik N., Williams R., Burns P. N. (2011). Investigation of vaporized submicron perfluorocarbon droplets as an ultrasound contrast agent. *Ultrasound in Medicine & Biology*.

[45] Giesecke T., Hynynen K. (2003). Ultrasound-mediated cavitation thresholds of liquid perfluorocarbon droplets in vitro. *Ultrasound in Medicine & Biology*.

[46] Kripfgans O. D., Fabiilli M. L., Carson P. L., Fowlkes J. B. (2004). On the acoustic vaporization of micrometer-sized droplets. *The Journal of the Acoustical Society of America*.

[47] Frost D. L. (1989). Initiation of explosive boiling of a droplet with a shock wave. *Experiments in Fluids*.

[48] Reznik N., Lajoinie G., Shpak O. (2014). On the acoustic properties of vaporized submicron perfluorocarbon droplets. *Ultrasound in Medicine & Biology*.

[49] de Jong N., Emmer M., Chin C. T. (2007). 'Compression-Only' behavior of phospholipid-coated contrast bubbles. *Ultrasound in Medicine & Biology*.

[50] Sheeran P. S., Wong V. P., Luois S. (2011). Decafluorobutane as a phase-change contrast agent for low-energy extravascular ultrasonic imaging. *Ultrasound in Medicine & Biology*.

[51] Kripfgans O. D., Fowlkes J. B., Miller D. L., Eldevik O. P., Carson P. L. (2000). Acoustic droplet vaporization for therapeutic and diagnostic applications. *Ultrasound in Medicine & Biology*.

[52] Kang S.-T., Lin Y.-C., Yeh C.-K. (2014). Mechanical bioeffects of acoustic droplet vaporization in vessel-mimicking phantoms. *Ultrasonics Sonochemistry*.

[53] Radhakrishnan K., Holland C. K., Haworth K. J. (2016). Scavenging dissolved oxygen via acoustic droplet vaporization. *Ultrasonics Sonochemistry*.

[54] Kang S.-T., Huang Y.-L., Yeh C.-K. (2014). Characterization of acoustic droplet vaporization for control of bubble generation under flow conditions. *Ultrasound in Medicine & Biology*.

[55] Thomas D. H., Sboros V., Emmer M., Vos H., De Jong N. (2013). Microbubble oscillations in capillary tubes. *IEEE Transactions on Ultrasonics, Ferroelectrics and Frequency Control*.

[56] Caskey C. F., Kruse D. E., Dayton P. A., Kitano T. K., Ferrara K. W. (2006). Microbubble oscillation in tubes with diameters of 12, 25, and 195 microns. *Applied Physics Letters*.

[57] Jamalian S., Lin S. T., Feldman C. Development of a branched microfluidic platform for acoustic quantification of microbubble populations.

[58] Lin S., Zhang G., Jamburidze A. (2018). Imaging of vaporised sub-micron phase change contrast agents with high frame rate ultrasound and optics. *Physics in Medicine and Biology*.

[59] Pajek D., Burgess A., Huang Y., Hynynen K. (2014). High-intensity focused ultrasound sonothrombolysis: the use of perfluorocarbon droplets to achieve clot lysis at reduced acoustic power. *Ultrasound in Medicine & Biology*.

[60] Kripfgans O., Orifici C., Carson P., Ives K., Eldevik O., Fowlkes J. (2005). Acoustic droplet vaporization for temporal and spatial control of tissue occlusion: a kidney study. *IEEE Transactions on Ultrasonics, Ferroelectrics and Frequency Control*.

[61] Zhang M., Fabiilli M. L., Haworth K. J. (2010). Initial investigation of acoustic droplet vaporization for occlusion in canine kidney. *Ultrasound in Medicine & Biology*.

[62] Carneal C. M., Kripfgans O. D., Carson P. L. (2011). A tissue-mimicking ultrasound test object using droplet vaporization to create point targets. *IEEE Transactions on Ultrasonics, Ferroelectrics and Frequency Control*.

[63] Burns P. N., Wilson S. R. (2007). Focal liver masses: enhancement patterns on contrast-enhanced images - concordance of US scans with CT scans and MR images. *Radiology*.

[64] Wang X., Hagemeyer C. E., Hohmann J. D. (2012). Novel single-chain antibody-targeted microbubbles for molecular ultrasound imaging of thrombosis. *Circulation*.

[65] Williams R., Wright C., Cherin E. (2013). Characterization of submicron phase-change perfluorocarbon droplets for extravascular ultrasound imaging of cancer. *Ultrasound in Medicine & Biology*.

[66] Matsunaga T. O., Sheeran P. S., Luois S. (2012). Phase-change nanoparticles using highly volatile perfluorocarbons: toward a platform for extravascular ultrasound imaging. *Theranostics*.

[67] Xu S., Zong Y., Li W., Zhang S., Wan M. (2014). Bubble size distribution in acoustic droplet vaporization via dissolution using an ultrasound wide-beam method. *Ultrasonics Sonochemistry*.

[68] Sheeran P. S., Matsunaga T. O., Dayton P. A. (2013). Phase-transition thresholds and vaporization phenomena for ultrasound phase-change nanoemulsions assessed via high-speed optical microscopy. *Physics in Medicine and Biology*.

[69] Sheeran P. S., Yoo K., Williams R., Yin M., Foster F. S., Burns P. N. (2017). More than bubbles: creating phase-shift droplets from commercially available ultrasound contrast agents. *Ultrasound in Medicine & Biology*.

[70] Singh R., Husseini G. A., Pitt W. G. (2012). Phase transitions of nanoemulsions using ultrasound: experimental observations. *Ultrasonics Sonochemistry*.

[71] Choudhury S. A., Xie F., Kutty S., Lof J., Stolze E., Porter T. R. (2018). Selective infarct zone imaging with intravenous acoustically activated droplets. *PLoS ONE*.

[72] Gao D., Gao J., Xu M. (2017). Targeted ultrasound-triggered phase transition nanodroplets for her2-overexpressing breast cancer diagnosis and gene transfection. *Molecular Pharmaceutics*.

[73] Mulvana H., Browning R. J., Luan Y. (2017). Characterization of contrast agent microbubbles for ultrasound imaging and therapy research. *IEEE Transactions on Ultrasonics, Ferroelectrics and Frequency Control*.

[74] Rojas J. D., Borden M. A., Dayton P. A. (2019). Effect of hydrostatic pressure, boundary constraints and viscosity on the vaporization threshold of low-boiling-point phase-change contrast agents. *Ultrasound in Medicine & Biology*.

[75] Guo S., Shi A., Xu S. (2017). Lowering of acoustic droplet vaporization threshold via aggregation. *Applied Physics Letters*.

[76] Tremblay-Darveau C., Sheeran P. S., Vu C. K. (2018). The role of microbubble echo phase lag in multipulse contrast-enhanced ultrasound imaging. *IEEE Transactions on Ultrasonics, Ferroelectrics and Frequency Control*.

[77] Samuel S., Duprey A., Fabiilli M. L., Bull J. L., Fowlkes J. B. (2012). In vivo microscopy of targeted vessel occlusion employing acoustic droplet vaporization. *Microcirculation*.

[78] Harmon J. S., Kabinejadian F., Seda R. Gas embolization in a rodent model of hepatocellular carcinoma using acoustic droplet vaporization.

[79] Wagland R., Richardson A., Ewings S. (2016). Prevalence of cancer chemotherapy-related problems, their relation to health-related quality of life and associated supportive care: a cross-sectional survey. *Supportive Care in Cancer*.

[80] Xu J., Tu H., Ao Z., Chen Y., Danehy R., Guo F. (2019). Acoustic disruption of tumor endothelium and on-demand drug delivery for cancer chemotherapy. *Nanotechnology*.

[81] Zhu L., Zhao H., Zhou Z. (2018). Peptide-functionalized phase-transformation nanoparticles for low intensity focused ultrasound-assisted tumor imaging and therapy. *Nano Letters*.

[82] Lipsman N., Meng Y., Bethune A. J. (2018). Blood–brain barrier opening in Alzheimer’s disease using MR-guided focused ultrasound. *Nature Communications*.

[83] Wu S.-Y., Fix S. M., Arena C. B. (2018). Focused ultrasound-facilitated brain drug delivery using optimized nanodroplets: Vaporization efficiency dictates large molecular delivery. *Physics in Medicine and Biology*.

[84] Sheeran P. S., Matsuura N., Borden M. A. (2017). Methods of generating submicrometer phase-shift perfluorocarbon droplets for applications in medical ultrasonography. *IEEE Transactions on Ultrasonics, Ferroelectrics and Frequency Control*.

[85] Rapoport N. (2012). Phase-shift, stimuli-responsive perfluorocarbon nanodroplets for drug delivery to cancer. *Wiley Interdisciplinary Reviews: Nanomedicine and Nanobiotechnology*.

[86] Marshalek J. P., Sheeran P. S., Ingram P., Dayton P. A., Witte R. S., Matsunaga T. O. (2016). Intracellular delivery and ultrasonic activation of folate receptor-targeted phase-change contrast agents in breast cancer cells in vitro. *Journal of Controlled Release*.

[87] Wang Y., Sui G., Teng D. (2019). Low intensity focused ultrasound (LIFU) triggered drug release from cetuximab-conjugated phase-changeable nanoparticles for precision theranostics against anaplastic thyroid carcinoma. *Biomaterials Science*.

[88] Cao Y., Chen Y., Yu T. (2018). Drug release from phase-changeable nanodroplets triggered by low-intensity focused ultrasound. *Theranostics*.

[89] Liu J., Shang T., Wang F. (2017). Low-intensity focused ultrasound (LIFU)-induced acoustic droplet vaporization in phase-transition perfluoropentane nanodroplets modified by folate for ultrasound molecular imaging. *International Journal of Nanomedicine*.

[90] Liu X., Zhao J., Guo D. (2015). Synthesis and evaluation of perfluorooctylbromide nanoparticles modified with a folate receptor for targeting ovarian cancer: In vitro and in vivo experiments. *International Journal of Clinical and Experimental Medicine*.

[91] Seo M., Williams R., Matsuura N. (2015). Size reduction of cosolvent-infused microbubbles to form acoustically responsive monodisperse perfluorocarbon nanodroplets. *Lab on a Chip *.

[92] Zhang X., Zheng Y., Wang Z. (2014). Methotrexate-loaded PLGA nanobubbles for ultrasound imaging and synergistic targeted therapy of residual tumor during HIFU ablation. *Biomaterials*.

[93] Crake C., Meral F. C., Burgess M. T., Papademetriou I. T., McDannold N. J., Porter T. M. (2017). Combined passive acoustic mapping and magnetic resonance thermometry for monitoring phase-shift nanoemulsion enhanced focused ultrasound therapy. *Physics in Medicine and Biology*.

[94] Zhang K., Li P., Chen H., Bo X., Li X., Xu H. (2016). Continuous cavitation designed for enhancing radiofrequency ablation via a special radiofrequency solidoid vaporization process. *ACS Nano*.

[95] Tempany C. M. C., McDannold N. J., Hynynen K., Jolesz F. A. (2011). Focused ultrasound surgery in oncology: overview and principles. *Radiology*.

[96] Xin Y., Zhang A., Xu L. X., Fowlkes J. B. (2018). The effects on thermal lesion shape and size from bubble clouds produced by acoustic droplet vaporization. *Biomedical Engineering Online*.

[97] Fuchs F. J. (2015). Ultrasonic cleaning and washing of surfaces. *Power Ultrasonics: Applications of High-Intensity Ultrasound*.

[98] Simon J. C., Sapozhnikov O. A., Kreider W., Breshock M., Williams J. C., Bailey M. R. (2018). The role of trapped bubbles in kidney stone detection with the color Doppler ultrasound twinkling artifact. *Physics in Medicine and Biology*.

[99] Smolock A. R., Cristescu M. M., Vlaisavljevich E. (2018). Robotically assisted sonic therapy as a noninvasive nonthermal ablation modality: proof of concept in a porcine liver model. *Radiology*.

[100] Vlaisavljevich E., Aydin O., Durmaz Y. Y. (2016). Effects of droplet composition on nanodroplet-mediated histotripsy. *Ultrasound in Medicine & Biology*.

[101] Vlaisavljevich E., Aydin O., Lin K.-W. (2015). The role of positive and negative pressure on cavitation nucleation in nanodroplet-mediated histotripsy. *Physics in Medicine and Biology*.

[102] Vlaisavljevich E., Aydin O., Yuksel Durmaz Y. (2015). Effects of ultrasound frequency on nanodroplet-mediated histotripsy. *Ultrasound in Medicine & Biology*.

[103] Bader K. B. (2018). The influence of medium elasticity on the prediction of histotripsy-induced bubble expansion and erythrocyte viability. *Physics in Medicine and Biology*.

[104] Aydin O., Vlaisavljevich E., Yuksel Durmaz Y., Xu Z., ElSayed M. E. H. (2016). Noninvasive ablation of prostate cancer spheroids using acoustically-activated nanodroplets. *Molecular Pharmaceutics*.

[105] Vlaisavljevich E., Durmaz Y. Y., Maxwell A., ElSayed M., Xu Z. (2013). Nanodroplet-mediated histotripsy for image-guided targeted ultrasound cell ablation. *Theranostics*.

[106] Ma M., Xu H., Chen H. (2014). A drug-perfluorocarbon nanoemulsion with an ultrathin silica coating for the synergistic effect of chemotherapy and ablation by high-intensity focused ultrasound. *Advanced Materials*.

[107] Tsochatzis E. A., Fatourou E., O'Beirne J., Meyer T., Burroughs A. K. (2014). Transarterial chemoembolization and bland embolization for hepatocellular carcinoma. *World Journal of Gastroenterology*.

[108] Farny C. H., Holt R. G., Roy R. A. (2009). Temporal and spatial detection of HIFU-induced inertial and hot-vapor cavitation with a diagnostic ultrasound system. *Ultrasound in Medicine & Biology*.

[109] Ryan K. L., D'Andrea J. A., Jauchem J. R., Mason P. A. (2000). Radio frequency radiation of millimeter wave length: potential occupational safety issues relating to surface heating. *Health Physics Journal*.

